# Lateral distal femoral condyle has more uniform cartilage wear in varus knee osteoarthritis

**DOI:** 10.1038/s41598-023-50168-3

**Published:** 2024-01-02

**Authors:** Maozheng Wei, Kuo Hao, Huijun Kang, Lingce Kong, Fei Wang

**Affiliations:** Department of Orthopaedic Surgery, Hebei Medical University Third Hospital, NO 139 Ziqiang Road, Shijiazhuang, 050051 Hebei People’s Republic of China

**Keywords:** Bone, Musculoskeletal system

## Abstract

Bone resection is highly valued in total knee arthroplasty (TKA), but how to determine the amount of distal femur resection is still controversial. The purpose of this study was to explore how to use lateral condyle as a reference for distal femoral osteotomy in TKA. Magnetic resonance imaging (MRI) and Radiographic images from 118 nonarthritic subjects and 123 osteoarthritis (OA) subjects were used to assess the cartilage wear pattern of the distal femur in varus knees. Measurements were performed on three-dimensional reconstruction after virtual bone cutting. The difference between the resection amount of distal (0°) and posterior (90°) was calculated when the medial condyle was used as a reference in OA patients. The osteotomy amount on lateral was calculated in nonarthritic subjects when the medial condylar osteotomy was consistent with the thickness of the implants. In 43% of OA patients, there was > 1 mm difference between the 0° and 90° in medial condyle cartilage, and no difference was observed in lateral. When using medial condyle as a reference for osteotomy, there was a difference of 1.3 ± 0.56 mm between the resection amount of 0° and 90°, and the difference was 0.24 ± 0.27 mm when using lateral condyle. Statistical analysis showed that there was a linear correlation between the resection amount of lateral condyle and mechanical lateral distal femoral angle (mLDFA) in nonarthritic subjects (*r* = 0.845, *p* < 0.001). Lateral distal femoral condyle has more uniform cartilage wear in varus knee osteoarthritis. Using the lateral condyle as the reference for distal femoral osteotomy is more suitable for the cartilage wear pattern of the varus knee. The position of cutting guide can be adjusted by preoperative measurements of mLDFA.

## Introduction

Total knee arthroplasty (TKA) is one of the most common and cost-effective operations for end-stage knee osteoarthritis (OA). However, the satisfaction rate of patients with TKA has only been 75 to 80%, mainly due to persistent pain and poor function^1^. The balance of soft tissue in TKA is affected by many factors, consisting of the looseness of surrounding soft tissue, the variation of bone geometry and the amount of bone cut. In Kinematic Alignment (KA) the thickness of bone cut should be the same as the implant to attain balanced soft tissue^2,3^.

Cartilage wear patterns were strongly influenced by limb alignment, the varus knees showing more loss in early flexion with thinner cartilage in medial compartments. When the knee joint is fully extended, the line parallel to the longitudinal axis of the femur intersects the best-fit circle on the peripheral boundary of the subchondral bone at 0°. Ninety-two percent of knees with varus deformity had > 1 mm cartilage wear at 0°on the medial femoral condyle and lateral femoral condyle had minor degeneration. The axis of motion of the knee joint crosses the center point of the medial and lateral condyles of the femur, Howell et al. found the subchondral bone of the medial and lateral femoral condyles has the same single radius of curvature^4,5^. In arthritic knees with varus alignment, the lateral femoral condyle had more native morphotype^4–7^. The lateral condyle of the distal femur has less cartilage and bone wear due to the pattern of cartilage wear in patients with varus knees. However, no study has investigated the possible effect of using the lateral condyle as a reference for distal femoral osteotomy.

This was a retrospective study conducted to provide anthropometric data by measuring the parameters of virtually resected distal femurs undergoing TKA using a 3D reconstruction process. The hypothesis of this study was that the lateral condyle of femur had more consistent cartilage wear at 0° and 90°. Osteotomy of the distal femur with the lateral condyle as a reference might reduce the cartilage thickness and lead to the difference in excision volume between the distal femur and the posterior condyle of femur.

## Method

A total of 123 knees from 112 OA patients undergoing TKA and 118 knees from 108 nonarthritic participants were included in the study between April 2020 and June 2021. All included subjects had varus knees. Lower limb alignment was assessed by the mechanical axes of the femur and tibia on full-length lower limb slices in the standing position. The inclusion criteria of arthritic group were: primary, degenerative and non-inflammatory knee OA with moderate to severe pain and failure of conservative treatment. The exclusion criteria are (1) previous knee surgery; (2) knee joint infection; (3) stiffness or ankylosis of hip joint; (4) bone loss or nerve function defect; (5) the need of highly restrictive prostheses; (6) severe knee joint deformity: knee joint flexion < 90°, flexion contracture > 20°, varus or valgus deformity > 10°. Patients in nonarthritic group consulted the orthopedic surgeon for a complaint unrelated to the cartilage wear, such as a slight soft-tissue injury or avulsion fracture. The nonarthritic subjects with cartilage wear were excluded by magnetic resonance imaging (MRI). The present study was approved by our Institutional Review Board, and informed consent was acquired from all subjects. All methods were performed in accordance with the relevant guidelines and regulations.

### MRI and Radiographic parameters

**Figure 1:**
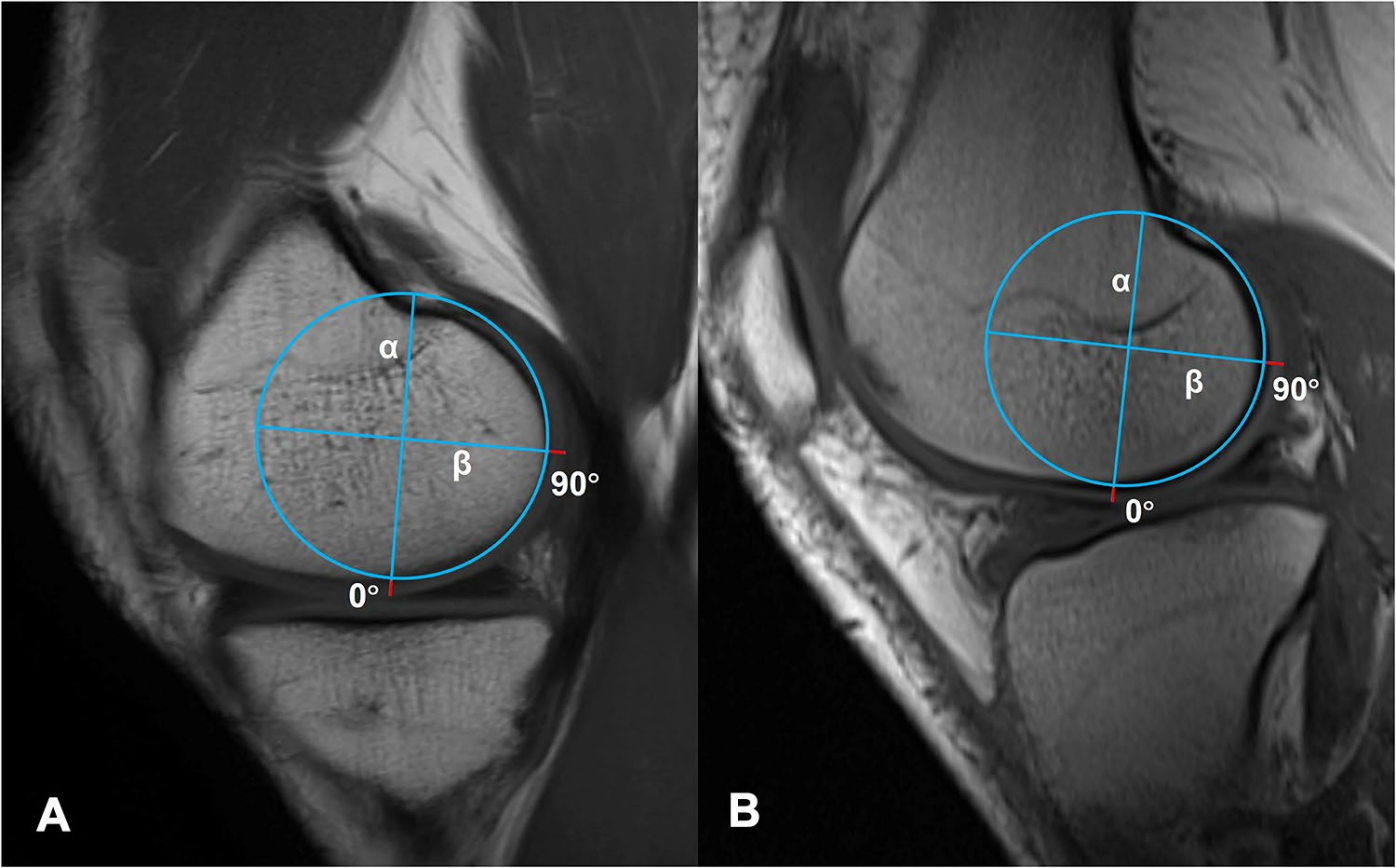
The method for measuring and computing cartilage thickness on the medial and lateral femoral condyle. (**A**) Medial condyle. (**B**) Lateral condyle. α is a line parallel to the longitudinal axis of the femur, and β is a line perpendicular to α. α and β are equally divided the best-fit circle on the peripheral boundary of the subchondral bone. The red lines are cartilage in contact with alpha and beta, representing 0° and 90° cartilage thickness, respectively.

All subjects of the study underwent full weight-bearing long-leg standing radiographs and lateral knee radiographs according to a standardized protocol to avoid bias. The MRI used for this study were obtained with a 1.5 T MRI (Sonata Magnetom, Siemens Medical Solutions, Erlagen, Germany) with the knee in or near full extension. Cartilage thickness was measured by applying the best-fit circle on the peripheral boundary of the subchondral bone. The thickness of the cartilage of each condyle was measured at 0° and 90°^5^ (Fig. 1). On MRI, we used previously described methods to determine the radius of a condyle and the thickness of the cartilage. The radius of a condyle was determined by drawing the best-fit circle of the subchondral cancellous bone interface of the femoral condyle in the sagittal image. In the sagittal plane, a clear boundary between the subchondral bone and the cartilage can be seen.

Computed tomography (CT) scans of the knees were obtained by a Philips CT (Philips Medical Systems, The Netherlands) and the acquired imagines were stored by the Picture Archiving and Communication System (PACS). RadiAnt-DICOM software (Medixant Ltd., Poznań, Poland), which has a mouse cursor that can automatically manifest distance and angle, was used to complete the measurements on the CT scans. To reduce the measurement error, two independent experienced orthopedic surgeons checked all of the images and measured relevant parameters in the case of double-blind.

**Figure 2:**
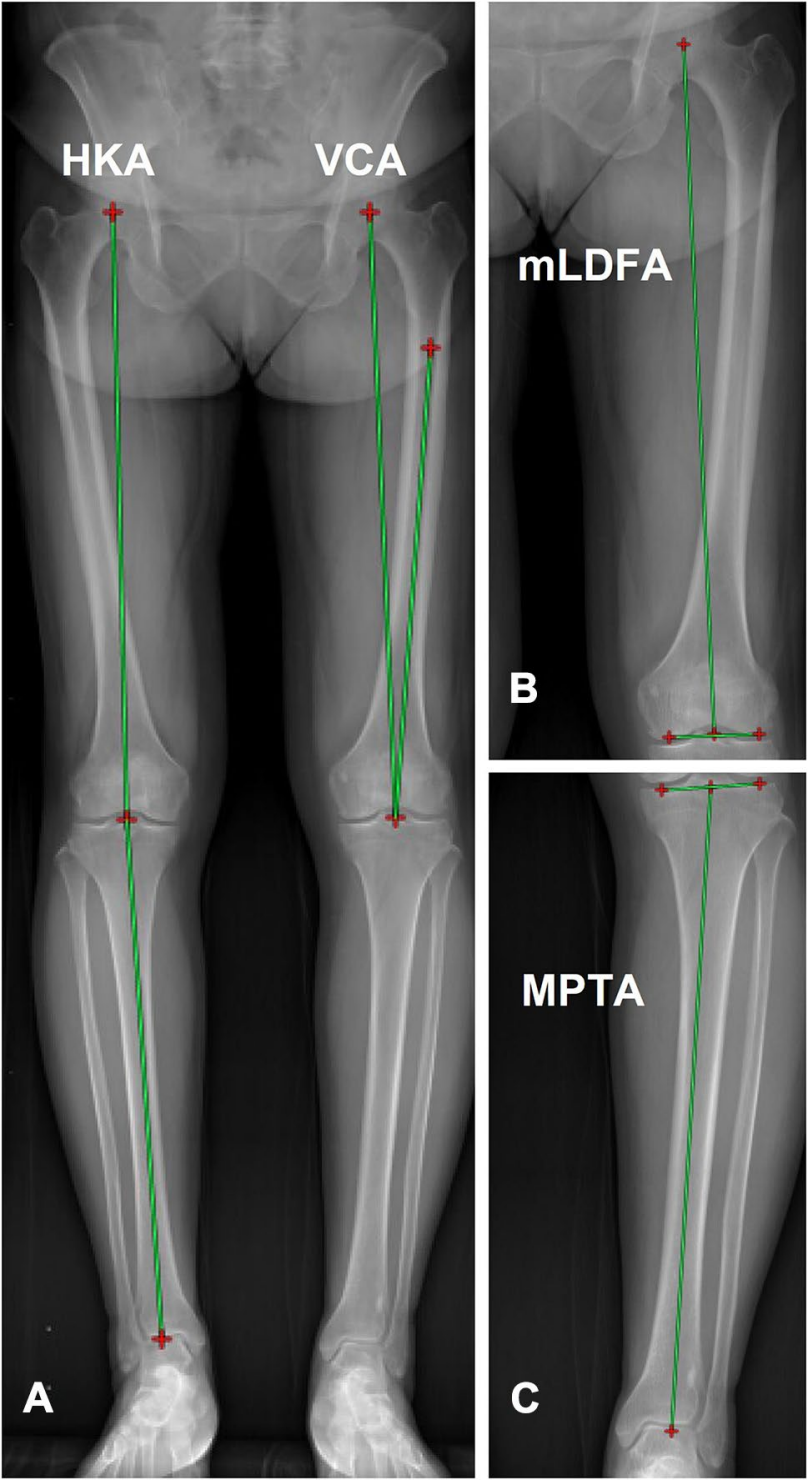
Radiographic measurements of coronal parameters. (**A**) Hip-knee-ankle angle (HKA) is angle between the line from the center of hip joint to the center of knee joint and the line from the center of knee joint to the center of ankle joint; Valgus correction angle (VCA) is the angle between the mechanical and anatomical axes of the femur. (**B**) Mechanical lateral distal femoral angle (mLDFA) is the lateral distal femoral angle between the femoral mechanical axis and the line tangential to the femoral condyles. (**C**) Medial proximal tibial angle (MPTA) is the medial angle between the tibial mechanical axis and the proximal tibial joint line connecting the distalmost points on the concavity of the subchondral bone of the medial and lateral tibial plateaus.

The following variables were measured^8,9^ and these were illustrated in Fig. 2.

I) HKA: Hip-knee-ankle angle. The angle between the line from the center of hip joint to the center of knee joint and the line from the center of knee joint to the center of ankle joint;
II) MPTA: Medial proximal tibial angle. The medial angle between the tibial mechanical axis and the proximal tibial joint line connecting the most distal points on the concavity of the subchondral bone of the medial and lateral tibial plateaus;
III) mLDFA: Mechanical lateral distal femoral angle. The lateral distal femoral angle between the femoral mechanical axis and the line tangential to the femoral condyles;
IV) VCA: Valgus correction angle. The angle between the mechanical and anatomical axes of the femur.

### 3D reconstruction and simulated osteotomy

Mimics 21.0 (Materialise, Leuven, Belgium) software was used for 3D reconstruction based on CT scans. Regional growth was performed to segment the femur, and each model was examined and corrected by a senior surgeon prior to measurement. The 3D model files were imported into 3-matic 13.0 (Materialise) for analysis.

**Figure 3:**
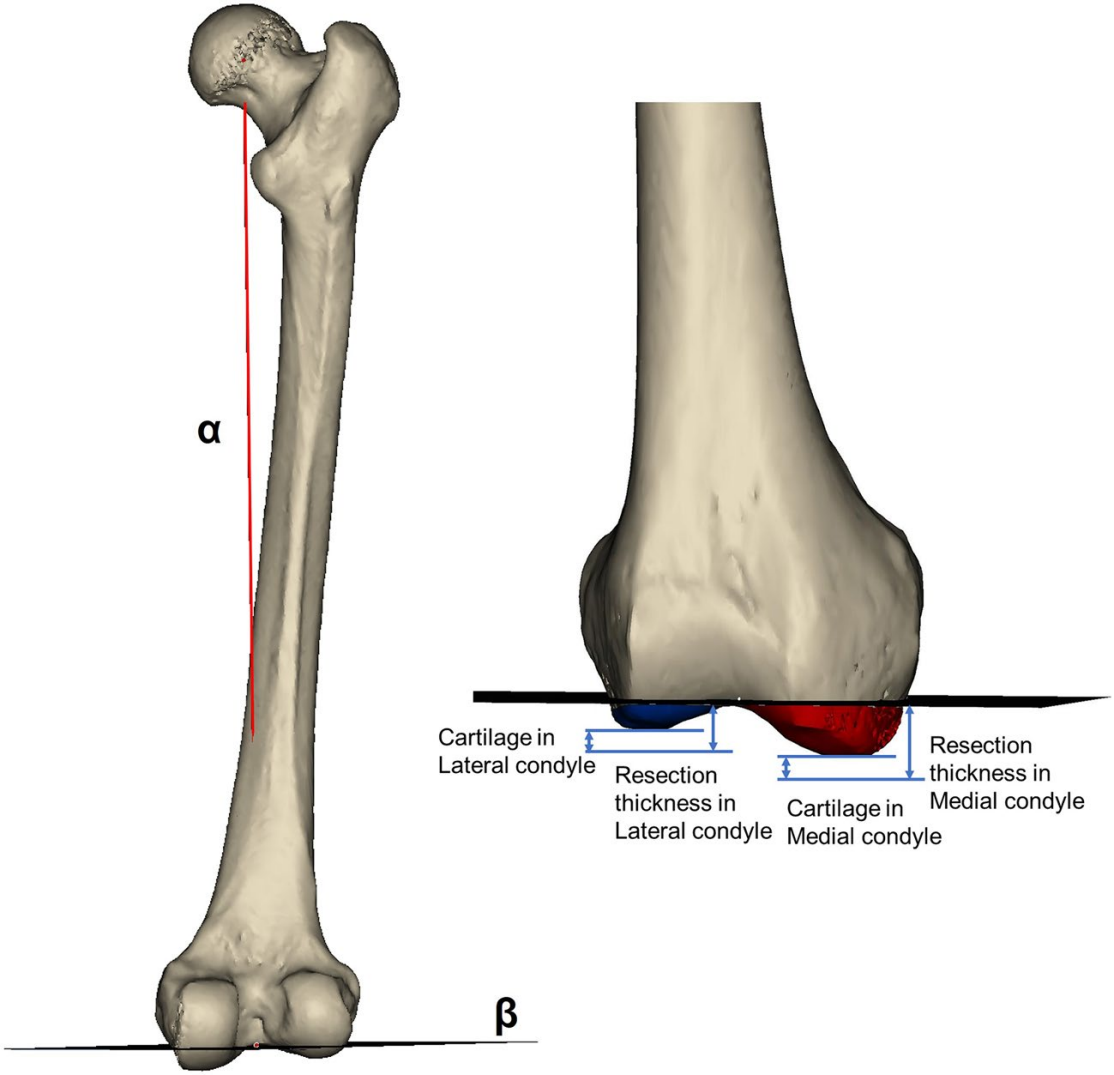
Simulated femoral resection. The cutting plane (β) was perpendicular to the mechanical axis (α). The thickness of distal femur resection was set to 8 mm, including the thickness of cartilage and bone. Cartilage thickness was consistent with the measurement by MRI.

A sphere was fitted to the surface of the femoral head, and the geometric center point of the sphere was defined as the center of the hip joint. The knee joint was centered at the midpoint of the femoral intercondylar fossa. The femoral mechanical axis was defined as a line connecting the center of the hip and the center of the knee^10^. The distal femoral and proximal tibial cuts were perpendicular to the mechanical axis. The thickness of distal femur resection was set to be consistent with the implant size of 8 mm, including the thickness of cartilage and bone (Fig. 3)^11^. Cartilage thickness was set to be consistent with the MRI date. When the lateral condyle was used as the reference, setting the resection depth at 8 mm from the distal lateral condyle. When the medial condyle was used as the reference, setting the resection depth at 8 mm from the distal medial condyle^12^.

### Statistical analysis

SPSS Statistics Package 21.0 (IBM, Armonk, New York, USA) was used to analyze data and *p* < 0.05 was defined as statistically significant. Scatterplots for each population were created to demonstrate alignment distributions for healthy and arthritic groups. The data were statistically analyzed by two researchers and all the measured variables and data are described as mean ± standard deviation. The paired-samples t-test was used to analyze the differences between measurement data and Levene's test was used to examine the homogeneity of the data. A linear regression test was performed to evaluate the correlation. The intra-class correlation values (ICC) were calculated to determine the reliability of inter-observer and intra-observer measurements.

### Ethical approval and consent to participate

The present study was approved by the Academic Ethics Committee of the Third Hospital of Hebei Medical University, and all patients provided their informed consent for participation and publication. All of the data and materials are available.

## Results

The demographic data of all patients, including gender, age, body mass index (BMI) and side, were collected before the operation and shown in Table 1. The ICC values of all measurement parameters were good to excellent, with the intra-observer ICCs and the interobserver ICCs ranging from 0.836 to 0.995 (Table 2).

**Table 1:** Demographic characteristics of the arthritic and nonarthritic groups.

Demographics	Arthritic	Nonarthritic
Participants (n)	112	107
Knees (n)	123	118
Age (years)	67.4 ± 7	31.3 ± 3.7
Sex		
Male	24% (27)	37% (40)
Female	76% (85)	63% (67)
BMI (kg/m^2^)	27.4 ± 3.6	21.8 ± 3.3
Side		
Left	52% (64)	41% (48)
Right	48% (59)	59% (70)
Radiographic parameters		
HKA (°)	171.79 (4.12)	177.26 (2.17)
MPTA (°)	85.15 (2.68)	85.54 (2.40)
mLDFA (°)	88.77 (2.30)	86.43 (2.37)

*BMI* body mass index, *HKA* hip-knee-ankle angle, *MPTA* medial proximal tibial angle, *mLDFA* mechanical lateral distal femoral angle.

Continuous variables are expressed as mean ± standard deviation and categorical variables are expressed as % (n).

**Table 2:** The inter-observer and intra-observer reliability of all measurements.

Parameter	Inter-observer		Intra-observer	
	ICC	95%CI	ICC	95%CI
Arthritic group				
Lateral femoral cartilage thickness at 0°	0.877	0.846–0.908	0.869	0.862–0.876
Medial femoral cartilage thickness at 0°	0.851	0.836–0.866	0.875	0.841–0.911
Lateral femoral cartilage thickness at 90°	0.887	0.863–0.911	0.87	0.843–0.896
Medial femoral cartilage thickness at 90°	0.919	0.911–0.926	0.861	0.843–0.878
HKA	0.980	0.971–0.985	0.990	0.985–0.994
MPTA	0.974	0.971–0.985	0.994	0.972–0.986
mLDFA	0.974	0.963–0.981	0.986	0.915–0.956
VCA	0.985	0.979–0.989	0.992	0.992–0.992
Nonarthritic group				
Lateral femoral cartilage thickness at 0°	0.864	0.852–0.876	0.898	0.86–0.935
Medial femoral cartilage thickness at 0°	0.888	0.865–0.913	0.875	0.847–0.903
Lateral femoral cartilage thickness at 90°	0.912	0.909–0.915	0.888	0.853–0.923
Medial femoral cartilage thickness at 90°	0.914	0.893–0.934	0.903	0.872–0.934
HKA	0.986	0.982– 0.990	0.990	0.986–0.997
MPTA	0.992	0.990–0.994	0.992	0.990–0.994
mLDFA	0.990	0.990–0.992	0.986	0.982–0.990
VCA	0.991	0.989–0.994	0.959	0.945–0.970

*ICC* intra-class correlation values; *HKA* hip-knee-ankle angle; *MPTA* medial proximal tibial angle; *mLDFA* mechanical lateral distal femoral angle; *VCA* valgus correction angle.

For varus arthritic knees, there was a difference in cartilage thickness between the distal and posterior condyles on the medial femoral condyle (*p* < 0.05). In the medial condyle, the cartilage thickness was 0.17 ± 0.31 mm at 0° and 1.08 ± 0.5 mm at 90°. No difference of cartilage thickness was observed in the lateral condyle between 0° and 90°. In the lateral condyle, the cartilage thickness was 1.23 ± 0.43 mm at 0° and 1.48 ± 0.47 mm at 90°. There was no specificity in the distribution of femoral cartilage in the medial and lateral femoral condyles in nonarthritic knees (Table 3).

**Table 3:** Cartilage thickness of the medial and lateral condyle at 0° and 90°.

	0°	90°	*P* value
Arthritic group			
Medial condyle (mm)	0.172 ± 0.311	1.093 ± 0.501	*P* < 0.001
Lateral condyle (mm)	1.232 ± 0.433	1.48 ± 0.47	*P* < 0.001
Nonarthritic group			
Medial condyle (mm)	1.838 ± 0.193	1.904 ± 0.216	0.004
Lateral condyle (mm)	1.893 ± 0.169	1.899 ± 0.18	0.950

The data was shown as mean ± standard deviation.

Three-dimensional reconstruction was used to simulate the osteotomy of osteoarthritis subjects perpendicular to the mechanical axis of the femur. When the medial condyle was used as the reference for distal femoral osteotomy, there was a difference of 1.3 ± 0.56 mm between the distal femoral end and posterior femoral condyle, and 43% of patients had a difference of more than 1 mm. When the lateral condyle was used as the reference for distal femoral osteotomy, the difference between distal femoral and posterior femoral condyle resection was 0.24 ± 0.27 mm (Table 4).

**Table 4:** Bone resection of the medial and lateral condyle at 0° and 90°.

	Medial condyle	Lateral condyle
Bone resection thickness		
0° (mm)	7.83 ± 0.311	6.77 ± 0.43
90° (mm)	6.90 ± 0.50	6.52 ± 0.47
*P* valve	*P* < 0.001	*P* < 0.001
Difference of bone		
Resection thickness between 0° and 90° (mm)	0.92 ± 0.49	0.24 ± 0.27
Knees of resection difference > 1 mm	46% (56)	1.6% (2)

The data was shown as mean ± standard deviation. Knees of resection difference > 1 are expressed as % (n).

The simulated osteotomy of nonarthritic subjects showed that when the resection amount of the medial distal femoral condyle was set to 8 mm, the resection amount of the lateral distal femoral condyle was 4.81 ± 1.76 mm. There was a linear correlation between the osteotomy thickness of the lateral condyle and LDFA (*r* = 0.845, *p* < 0.001, Table 5). The resection amount of the lateral distal femoral condyle be predicted from the equation: Osteotomy thickness =  − 49.237 + 0.625 * mLDFA (R^2^ = 0.715).

**Table 5:** Pearson correlation and *r*—value of the parameters.

Parameter	Osteotomy thickness of the lateral condyle, mm
HKA	− 0.028
MPTA	0.046
mLDFA	0.845*
VCA	0.107

*HKA* hip-knee-ankle angle; *MPTA* medial proximal tibial angle; *mLDFA* mechanical lateral distal femoral angle; *VCA* valgus correction angle.

*Statistically significant.

## Discussion

In this study, it was found that Lateral distal femoral condyle has more uniform cartilage wear in varus knee osteoarthritis. Based on this, using the lateral femoral condyle as the reference for distal femoral osteotomy can make the bone resection in the distal femur and the posterior femoral condyle more consistent. For the unworn knee, it was found that there was a correlation between mLDFA and osteotomy thickness of the lateral condyle, using the medial condyle as a reference. The surgeon can adjust the cutting guide according to the preoperative measured mLDFA and the thickness of the lateral condyle to obtain a more consistent resection of the distal femur and posterior femoral condyle.

Precise TKA surgical planning should take into account the pattern of cartilage wear in arthritic patients with varus knees. Residual cartilage in patients with osteoarthritis may affect the accuracy of the osteotomy. Nam et al. proposed surgical planning for TKA based on CT does not consider articular cartilage and could lead to external malrotation of the femoral implant^13^.

The femoral cartilage wear is strongly associated with lower extremity mechanical alignment. MRI measurements of the knee in arthritic patients with varus knees showed that there were 43% of patients with cartilage thickness differences > 1 mm between 0° and 90° of medial femoral condyles, consistent with previous reports. The progression of arthritis is accompanied by the wear of femoral cartilage. Johnson et al. found the wear of femoral cartilage is affected by the lower limb alignment through femorotibial cartilage maps and joint kinematics. Cartilage wear of varus knee is mainly concentrated in the medial compartment of early flexion, while valgus knee has more cartilage wear in the posterolateral femoral condyle during deep flexion^4^. Denis et al. found that 92% of OA patients with varus knee had cartilage wear of more than 1 mm at 0° flexion, while the lateral femoral condyle had minor cartilage degeneration. Morphological studies found the subchondral bone of the medial and lateral femoral condyles has the same single radius of curvature and a single transverse axis^5,7,14^. There was greater cartilage wear on the medial condyle of the distal femur and more asymmetry wear between 0° and 90° than the lateral. Therefore, we recommend using the lateral femoral condyle as a reference for distal femoral osteotomy.

Cartilage thickness in the medial condyle of 0° was found to be significantly less than that on 90° in OA patients with varus knees (Table 3), using the medial condyle as a reference resulted in an increase in the radius of curvature in extension. 11% to 26% revision TKAs were performed for instability while part of them had mid-flexion instability^15^. Clary et al. identify the sudden reduction in the radii-of-curvature of femoral condyle as a potential cause of clinically observed paradoxical anterior femoral translation in mid-flexion^16^.

Different femoral sagittal design in TKA were based on different theories. Multi-radius (MR) design was based on the theory of knee rotation center which was first proposed by Frankel et al. in 1971. It reported that knee flexion occurs around a varying transverse axis, the flexion axis varies in a helical fashion during the flexion process, and the instantaneous rotation center of the femoral posterior condyle forms a “J curve^17,18^’’. Single-radius (SR) design had a uniform radius of curvature. It designed based on the principle that superficial medial collateral ligament is isometric throughout its range of movement. SR regards the femoral condyle as a spherical sphere with a single radius^19^. SR had theoretical advantages, and it provided a more posterior flexion axis and a longer extensor moment arm and maintained stabilization during movement, especially in mid-flexion^20^. SR design required the radius of curvature in the sagittal plane. No difference in cartilage wear was observed between the distal and posterior condyles of the lateral femoral condyle. Using the lateral femoral condyle as the reference for distal femoral osteotomy has the advantage of keeping the radius of curvature consistent when applying the SR design implants.

**Figure 4:**
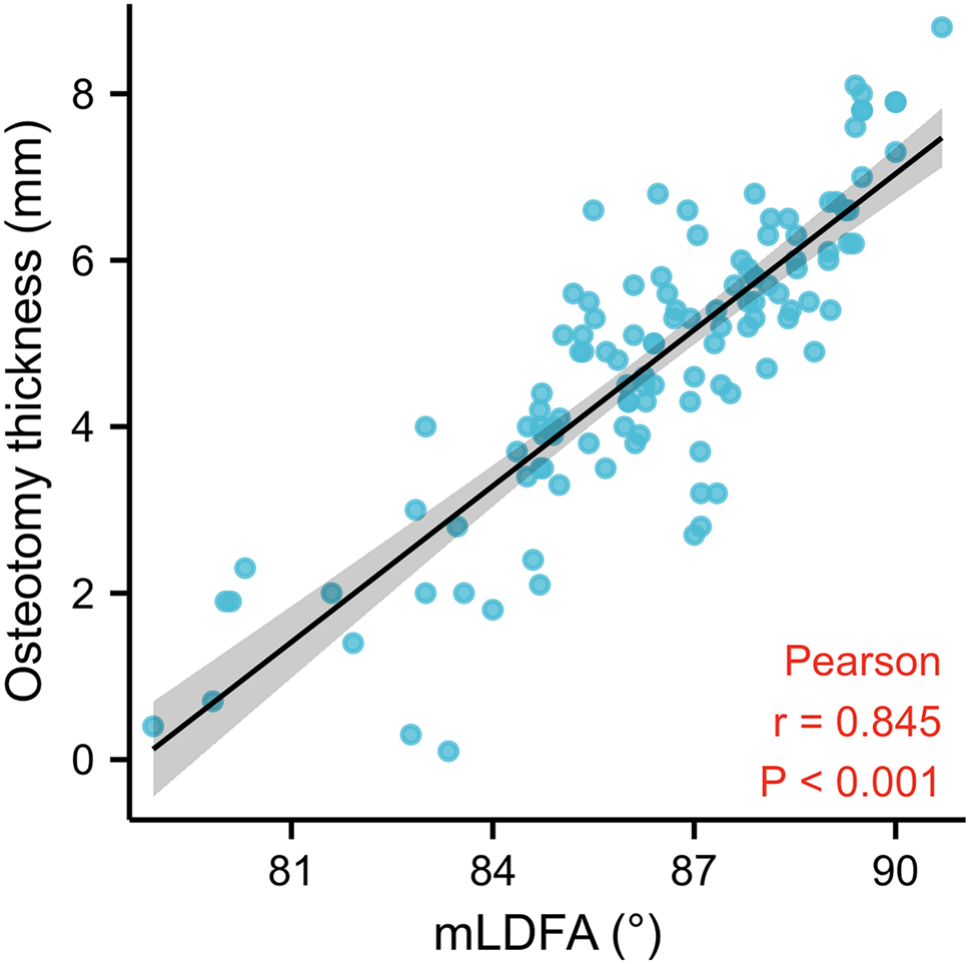
Scatterplot of the osteotomy thickness versus the mechanical lateral distal femoral angle (mLDFA). The black line indicates the linear correlation between the two variables.

When the lateral condyle was used as a reference, the resection amount of the distal femur was affected by the geometric shape of the femur, and the position of the osteotomy line of the distal femur should be adjusted by morphological measurement of the femur. In this study, multiple linear regression analysis was performed on the resection amount measured by simulated osteotomy in nonarthritic subjects. mLDFA was linearly correlated with the resection amount differences between the medial and lateral condyles at 0° (*r* = 0.845, *p* < 0.001), and a 0.85 mm increment of osteotomy thickness on lateral condyle was observed with every 1° mLDFA increment when the osteotomy of the medial condyle was set at 8 mm (Fig. 4). Using Fig. 4 and the equation: Osteotomy thickness =  − 49.237 + 0.625 * mLDFA, the surgeon can predict the resection thickness of lateral condyle by measuring mLDFA preoperatively and use this information intra-operatively. The amount of osteotomy may not affect limb alignment but has important implications for ligament tension, tissue balance and clinical outcome^16^. Many scholars attributed the unsatisfactory results after TKA to the inability of knee prosthesis to simulate physiological and natural knee motion and suggested to amend the surgical strategy^20,21^. Severe deformities lead to elevated joint lines. The least worn part of the joint was often used as a reference for bone cuts, and the wear of bone and cartilage in the medial condyle might result in over-resection^22^. Mullaji et al. proposed that the thickness of distal femoral resection should be determined according to the degree of bone defect in the medial femoral condyle^23^. Denis et al. suggested referencing guide should be adjusted to compensate for the mean 1.9 mm cartilage wear at the distal medial condyle^5^. Yue et al. proposed the intercondylar notch ceiling could be used as an accurate landmark to determine the proper distal femoral resection level during TKA^10^.

There were some limitations in this study. First, the nonarthritic group for the image analysis was selected for patients with slight soft-tissue injury or avulsion fracture rather than patients without knee joint disease. The nonarthritic subjects with cartilage wear were excluded by MRI, but there may be potential effects. Second, a relatively small sample size for each subject group, this was due mainly to the relatively lengthy segmentation process. Third, it was a retrospective study with non-randomized design, which had inherent drawbacks.

## Conclusion

With the wear patterns of cartilage in varus osteoarthritic knees treated with TKA, it showed significantly more wear at 0° in the medial compartment than at 90°. Distal femoral resection using medial condyle as reference resulted in > 1 mm difference of resection between 0° and 90°of flexion. The lateral femoral condyle has more consistent cartilage wear. Orthopedic surgeons can adjust the reference position according to the preoperative measured mLDFA and the measured thickness of the lateral condyle to obtain a more balanced resection in the distal femur and posterior femoral condyle.

## Data Availability

The datasets used or analyzed during the current study are available from the corresponding author on reasonable request.

## Abbreviations

TKA: Total knee arthroplasty
OA: Osteoarthritis
mLDFA: Mechanical lateral distal femoral angle
MRI: Magnetic resonance imaging
CT: Computed tomography
ICC: Intra-class correlation values
BMI: Body mass index
MR: Multi-radius
SR: Single-radius
